# Organohalide respiration potential in marine sediments from Aarhus Bay

**DOI:** 10.1093/femsec/fiac073

**Published:** 2022-06-11

**Authors:** Chen Zhang, Siavash Atashgahi, Tom N P Bosma, Peng Peng, Hauke Smidt

**Affiliations:** Laboratory of Microbiology, Wageningen University & Research, Stippeneng 4, 6708 WE Wageningen, The Netherlands; Biotechnology Research Institute, Chinese Academy of Agricultural Sciences, Beijing 100081, PR China; Laboratory of Microbiology, Wageningen University & Research, Stippeneng 4, 6708 WE Wageningen, The Netherlands; Laboratory of Microbiology, Wageningen University & Research, Stippeneng 4, 6708 WE Wageningen, The Netherlands; Laboratory of Microbiology, Wageningen University & Research, Stippeneng 4, 6708 WE Wageningen, The Netherlands; Department of Civil and Environmental Engineering, University of Michigan, Ann Arbor, Michigan 48109-2125, United States; Laboratory of Microbiology, Wageningen University & Research, Stippeneng 4, 6708 WE Wageningen, The Netherlands

**Keywords:** Aarhus Bay marine sediments, tetrachloroethene (PCE), 2,6-Dibromophenol (2,6-DBP), reductive dehalogenation, organohalide respiring prokaryotes, 16S rRNA gene amplicon sequencing

## Abstract

Organohalide respiration (OHR), catalysed by reductive dehalogenases (RDases), plays an important role in halogen cycling. Natural organohalides and putative RDase-encoding genes have been reported in Aarhus Bay sediments, however, OHR has not been experimentally verified. Here we show that sediments of Aarhus Bay can dehalogenate a range of organohalides, and different organohalides differentially affected microbial community compositions. PCE-dechlorinating cultures were further examined by 16S rRNA gene-targeted quantitative PCR and amplicon sequencing. Known organohalide-respiring bacteria (OHRB) including *Dehalococcoides*, *Dehalobacter* and *Desulfitobacterium* decreased in abundance during transfers and serial dilutions, suggesting the importance of yet uncharacterized OHRB in these cultures. Switching from PCE to 2,6-DBP led to its complete debromination to phenol in cultures with and without sulfate. 2,6-DBP debrominating cultures differed in microbial composition from PCE-dechlorinating cultures. *Desulfobacterota* genera recently verified to include OHRB, including *Desulfovibrio* and *Desulfuromusa*, were enriched in all microcosms, whereas *Halodesulfovibrio* was only enriched in cultures without sulfate. Hydrogen and methane were detected in cultures without sulfate. Hydrogen likely served as electron donor for OHR and methanogenesis. This study shows that OHR can occur in marine environments mediated by yet unknown OHRB, suggesting their role in natural halogen cycling.

## Teaser

Eco-physiological characterization of organohalide respiration from Aarhus Bay marine sediments.

## Introduction

Halogenated organic compounds, also termed organohalides, can be man-made or of natural origin. Over 5000 organohalides with a natural origin have been reported (Gribble 1996, 2012, 2015). Marine environments are a large reservoir of natural organohalides, which are produced via biotic and abiotic mechanisms (Gribble 1996, 2012, 2015). Marine organisms such as algae, sponges, corals and microorganisms recruit either substrate-specific halogenases or haloperoxidases to catalyse the biotic formation of organohalides (Atashgahi *et al*. 2018, Bayer *et al*. 2013, Gribble 2015, Gutleben *et al*. 2019, Ozturk *et al*. 2013, Wagner *et al*. 2009, Wever and van der Horst 2013). Abiotic halogenation on the other hand occurs by photochemical reaction, volcanic eruption and Fenton-like mechanisms (Comba *et al*. 2015, Leri *et al*. 2014, Méndez-Díaz *et al*. 2014). Some of these natural organohalides have excellent medical potential to cure cancers, and viral- and bacterial infections (Gribble 2015), whereas some are toxic such as polychlorinated dibenzo-p-dioxin (PCDDs) and polybrominated diphenyl ethers (PBDEs) (Wiseman *et al*. 2011). Further, most of the highly halogenated natural organohalides, for example PBDEs, have been found precipitated and buried in marine sediments of millions of years of age (Hashimoto *et al*. 1995, Moon *et al*. 2007). Accordingly, anaerobic dehalogenation likely occurs in marine sediments as the biggest anoxic environment on Earth, and thus contributing to the detoxification, decomposition and recycling of toxic/persistent organohalides (Ahn *et al*. 2003, Häggblom *et al*. 2003, Liu *et al*. 2017, Peng *et al*. 2020a).

Halides are replaced by hydrogen through reductive dehalogenation that often is linked to a specific type of anaerobic respiratory metabolism termed organohalide respiration (OHR). In this process, organohalides serve as the terminal electron acceptors to conserve energy (Dolfing and Tiedje 1987, Mohn and Kennedy 1992, Smidt and De Vos 2004). This process has been documented in bacteria known as organohalide-respiring bacteria (OHRB) (Atashgahi *et al*. 2016, Fincker and Spormann 2017, Smidt and De Vos 2004). OHR is mediated by reductive dehalogenase (RDase) enzymes (Adrian and Loeffler 2016, Fincker and Spormann 2017). Since the isolation of the first OHRB, *Desulfomonile tiedjei* (Dolfing and Tiedje 1987, Mohn and Tiedje 1990), a broad diversity of OHRB have been isolated, belonging to three phyla: *Chloroflexi*, *Firmicutes*, and Proteobacteria (Atashgahi *et al*. 2016, Fincker and Spormann 2017, Türkowsky *et al*. 2018). Members of the genera *Dehalococcoides* (*Dhc*) and *Dehalogenimonas* (*Dhg*) within the *Chloroflexi*, and *Dehalobacter* (*Dhb*) in the *Firmicutes* are obligate OHRB based on their restriction to OHR as the sole energy metabolism (Fincker and Spormann 2017, Holscher *et al*. 2004, Maillard *et al*. 2003, Moe *et al*. 2009, Molenda *et al*. 2016, Muller *et al*. 2004). Members of *Desulfitobacterium* (*Dsb*) in *Firmicutes*, *Sulfurospirillum* (*Sul*) in *Epsilonproteobacteria* and various *Desulfobacterota* (previously *Deltaproteobacteria*) species including members of *Geobacter* (*Geo*), *Desulfoluna* and *Desulfovibrio* are considered as facultative OHRB that have a versatile metabolism including but not restricted to OHR (Liu and Haggblom 2018, Maphosa *et al*. 2010, Peng *et al*. 2020a). Intriguingly, up to 10% of all available *Desulfobacterota* genomes were found to contain at least one putative RDase gene (Liu and Haggblom 2018). Accordingly, a recent genome-guided study experimentally verified OHR potential in three *Desulfobacterota* isolates (Liu and Haggblom 2018).

Using degenerate primers based on sequences of well-characterized RDase genes, different RDase genes have been reported from subsea sediments of the Pacific Ocean close to Peru, Japan, Oregon (United States) and the eastern equator, that were diverse and phylogenetically distinct from their characterised counterparts from contaminated terrestrial environments (Futagami *et al*. 2009, Futagami *et al*. 2013). Application of various techniques such as (meta)genomics and (meta)transcriptomics have revealed an enormous diversity of RDase genes in marine sediments (Jochum *et al*. 2017, Jochum *et al*. 2018, Petro *et al*. 2019a, Petro *et al*. 2019b, Zinke *et al*. 2017). For instance, meta-transcriptomic analysis of deep subsea sediments from site M59, close to Aarhus Bay, in the Baltic sea led to the discovery of a trichloroethene (TCE) reductive dehalogenase (*tceA*)-like gene that was transcribed, indicating the potential for PCE or TCE dechlorination in deep sea sediments as previously described for other environments (Fung *et al*. 2007, Magnuson *et al*. 1998, Zinke *et al*. 2017). Recently, single-cell genomics and metagenomics analyses revealed the presence of RDase genes in sulfate-rich sediments of Aarhus Bay (Fincker *et al*. 2020, Jochum *et al*. 2018). This begged the question whether there is actual OHR potential in marine sediments of Aarhus Bay that may prevent accumulation of organohalides and contribute to halide and CO_2_ recycling back to the sea. It is interesting to note that sulfate reduction has been reported to be the predominant bioprocess in near-surface Aarhus Bay marine sediments (Leloup *et al*. 2009, Ozuolmez,*et al*. 2020, Petro *et al*. 2019b), in which reductive dehalogenation is likely to be inhibited by the produced and accumulated sulfide according to previous reports (Azizian *et al*. 2010b, DeWeerd *et al*. 1991, Mao *et al*. 2017, Nelson *et al*. 2002). Henceforth, taking the presence and absence of sulfate into consideration would add more insights to explore OHR potential in laboratory microcosms.

In this study, we showed that cultures obtained from marine sediments from Aarhus Bay were capable of dechlorinating, debrominating and deiodinating organohalides, further corroborating above-mentioned (meta)genome based studies with respect to the predicted occurrence of OHR in marine sediments. Interestingly, the microbial communities in reductively dechlorinating and debrominating enrichment cultures were divergent, suggesting the coexistence of diverse OHRB. Furthermore, enriched microorganisms did not belong to the well-identified OHRB, suggesting the presence of novel dehalogenators in these pristine marine environments.

## Materials and Methods

### Chemicals

PCE, TCE, cDCE, *trans*-dichloroethene (tDCE) vinyl chloride (VC), ethene (ETH), 2,4,6-trichlorophenol (2,4,6-TCP), 2,6-dichlorophenol (2,6-DCP), 2,4- dichlorophenol (2,4-DCP), chlorophenol (CP), 2,6-DBP, 3-bromophenol (3-BP), 2-bromophenol (2-BP), 2,4,6-triiodophenol (2,4,6-TIP), 2,6-diiodophenol (2,6-DIP), 2,4-diiodophenol (2,4-DIP), 2-iodophenol (2-IP) and 4-iodophenol (4-IP), phenol, 1,4-dibromobenzene (1,4-DBB), bromobenzene (BB), and benzene were purchased from Sigma-Aldrich. Sulfate (0.5 M) and lactate (0.5 M) stock solutions were prepared by filter sterilization (syringe filter, 0.2 µm, mdimembrane, Ambala Cantt, India). All other (in)organic chemicals were of analytical grade.

### Sediment collection and enrichment set-up

**Figure 1.**
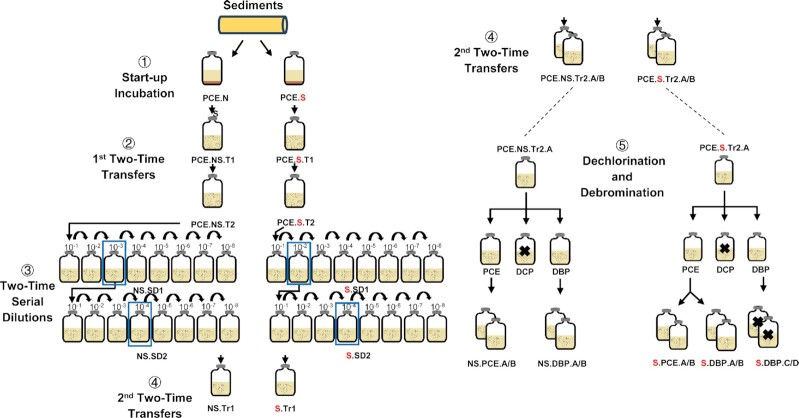
Experimental outline of reductive dehalogenation by marine sediments from Aarhus Bay M5 station. This experiment was carried out in 5 steps as labelled by ①, ②, ③, ④ and ⑤. “Sediments” stands for samples from near-surface marine sediments (3-35cm) of Aarhus Bay. Transfer volumes are 10% (v/v) in steps ①, ②, and ③, and 5% (v/v) in steps ④ and ⑤. Curved arrows in step ③ indicate the transfer pattern in 10 fold dilutions. NS: sulfate-free; S in red: sulfate-amended; Blue boxes in serial dilutions indicate the highest dilution at which dechlorination of PCE to cDCE was observed. Consequently, these are also the cultures that were used for the subsequent transfer; Bold cross marks indicate cultures that were unable to dehalogenate DCP or DBP.

Samples were kindly provided by Kasper U. Kjeldsen (Aarhus University). Specifically, samples were collected from a marine sediment core at station M5 in Aarhus Bay (56.103333 N, longitude 10.457833 E), Denmark, and were depth-fractioned into two parts: 3–35 cm and 36–68 cm. Ten grams of sediment from each fraction was transferred into 120 mL serum bottles containing 50 mL anoxic marine medium as previously described (Monserrate and Häggblom 1997, Peng *et al*. 2020a). Na_2_S·9H_2_O (0.48 g/L, 2 mM) was added as reducing reagent and Resazurin (0.005 g/L) as redox indicator. The bottle headspace was exchanged with N_2_ and CO_2_ (80 : 20%, 140 KPa), bottles were sealed with Teflon-coated butyl rubber septa and aluminum crimp caps (GRACE, MD, USA) and incubated statically in the dark at 20°C. Some organohalides were added to the marine medium separately and tested, including PCE, 2,6-DBP, 1,4-DBB, 3-BP, 2,4,6-TCP, and 2,4,6-TIP with/without additional 5mM sulfate and 5mM lactate as the electron donor. Subsequently, experiments focused first on more detailed characterization of PCE dechlorination. The dechlorinating cultures were spiked with PCE (100 µM) as electron acceptor and lactate (5 mM) serving as the electron donor and carbon source. One set of bottles received sulfate (5 mM) as an additional electron acceptor (designated “S”; bottles with only PCE were designated “NS”) (Fig. 1). Two transfers were set as the initial step to enrich for PCE dechlorinating microorganisms. Cultures were transferred when the PCE was reductively dechlorinated to cDCE. For each transfer, 10% (v/v) of the mother cultures was transferred to fresh medium (Fig. 1), with cultures being designated PCE.NS.T1 and PCE.S.T1 for first transfers, and PCE.NS.T2 and PCE.S.T2 indicating the second transfer. Second transfers were subsequently 10-fold serially diluted (SD) according to the dilution-to-extinction principle (NS.SD1, S.SD1; Fig. 1). Highest dilutions for which dehalogenation was observed (NS.SD13, S.SD12, indicated by blue boxes in Fig. 1) were used as inocula for a second series of 10-fold serial dilutions (NS.SD2, S.SD2; Fig. 1). Highest dilutions with active dehalogenation (NS.SD24, S.SD24; Fig. 1) after four spikes of 250 µM PCE and corresponding chemicals (5 mM lactate in NS, 5 mM lactate and 5 mM sulfate in S cultures) in the second serial dilution were used to inoculate a new set of transfers (NS.Tr1 and S.Tr1, Fig. 1). These cultures were transferred once more to obtain duplicate sediment-free enrichment cultures (PCE.NS.Tr2.A/B and PCE.S.Tr2.A/B, Fig. 1). In addition to dechlorination of the aliphatic organohalide PCE, the potential to dehalogenate aromatic organohalides was also investigated. To this end, 2,6-DBP and 2,6-DCP were selected as representative electron acceptors and injected into fresh cultures separately, to which microbes were transferred from sediment-free PCE dechlorinating enrichments (PCE.NS.Tr2.A and PCE.S.Tr2.A). In order to obtain a comprehensive overview of PCE dechlorination and 2,6-DBP debromination potential under both sulfate-free and sulfate-amended conditions, the test cultures (PCE and DBP) were further transferred to duplicate cultures (NS.PCE.A/B, NS.DBP.A/B, S.PCE.A/B and S.DBP.C/D). Due to the lack of debromination from 2,6-DBP under sulfate-amended conditions (S.DBP.C/D), a new set of duplicate cultures (S.DBP.A/B) was inoculated from the PCE culture (Fig. 1). During the 5^th^ part of the experiment “Dechlorination and Debromination”, duplicates of dechlorinating cultures were spiked five times with PCE (250 µM) and in the same way, debrominating cultures were also spiked five times with 2,6-DBP (100 µM). For the duplicate 2,6-DBP debrominating cultures, additional lactate (5 mM) and lactate/sulfate (5 mM) were also injected into the cultures from the second spike, based on the assumption that debromination was halted due to depletion of lactate. Before each spike, hydrogen and methane were measured in headspace samples, and 2 ml culture was sampled and centrifuged for 5 min at 8000 *g*. Supernatants were used for metabolite measurements, whereas pellets were used for DNA extraction.

### Chemical analyses

Gas chromatography combined with mass spectrometry (GC-MS) was used to measure PCE, TCE and cDCE using an Rt^®^-Q-BOND column (Retek, PA, USA) and a DSQ MS (Thermo Fisher Scientific). Helium served as carrier gas with a flow rate of 2 ml/min. The split ratio was 30 and the inlet temperature was 100°C. The temperature program included 40°C holding for 1 min, followed by an increase by 40°C/min to 260°C and a final hold at this temperature for 1.5 min. Hydrogen and methane were detected by a Compact GC (Global Analyzer Solutions, Breda, The Netherlands) with a pulsed discharge ionization detector (GC-PDD). Halogenated phenols, benzenes, benzene and phenol were measured using a Thermo Scientific Accela High-performance liquid chromatography (HPLC) system equipped with an Agilent Poroshell 120 EC-C18 column and a UV/Vis detector (set at 210 nM for aromatic halogenated compounds). Short chain fatty acids were measured using a SHIMADZU LC2030 PLUS coupled with a Shodex SUGAR Series^®^ SH1821 column. Sulfate was analysed by using a Thermo Scientific Dionex™ ICS-2100 Ion Chromatography System (Dionex ICS-2100). Sulfide was measured by a photometric method using methylene blue as described previously (Cline 1969).

### Genomic DNA isolation and quantitative PCR (qPCR)

Genomic DNA was isolated from pelleted biomass using the DNeasy PowerSoil Kit (QIAGEN, Hilden, Germany) following manufacturer's instructions. qPCR was used to quantify the copy number of 16S rRNA genes with primers targeting total bacteria (Muyzer *et al*. 1993), and well-characterized OHRB, including *Dehalococcoides* (Smits *et al*. 2004), *Dehalogenimonas* (Chen *et al*. 2013), *Dehalobacter* (Smits *et al*. 2004), *Desulfitobacterium* (Smits *et al*. 2004), *Geobacter* (Amos *et al*. 2007) and *Sulfurospirillum* (Sutton *et al*. 2015). Furthermore, primers targeting well-characterized functional RDase genes *tceA*, *vcrA*, and *bvcA* were used as listed in Table S1. All reactions were performed in triplicate using a C1000 Thermal Cycler (CFX384 Real-Time system, Bio-Rad Laboratories, Hercules, CA, USA) with iQ^TM^ SYBR Green Supermix (Bio-Rad Laboratories, Hercules, CA, USA) as outlined previously (Peng *et al*. 2019).

### Analysis of microbial composition based on 16S rRNA gene amplicon sequences

Barcoded amplicons of 16S rRNA genes were amplified targeting the V4 region of prokaryotic 16S rRNA genes. The forward primer 515F (5’- GTGCCAGC[AC]GCCGCGGTAA-3’) and reverse primer 806R (5’-GGACTAC[ACT][ACG]GGGT[AT]TCTAAT-3’) (Caporaso *et al*. 2011, Walters *et al*. 2016) were amended at the 5’-end with sample-specific barcodes. The PCR mixture (50 µL) was prepared containing 20 µL 5 × HF Green buffer (Thermo Fisher Scientific, the Netherlands), 1 μl (2 U) of Phusion hot start II High-Fidelity DNA polymerase (Thermo Fisher Scientific), primer mix (500 nM for each forward and reverse primer), and 500 nM dNTP (Promega, USA), 10 ng DNA template and nuclease-free water (Promega, USA). The PCR conditions were: 98°C, 30 s for pre-denaturation, followed by 25 cycles of 98°C, 10 s for denaturation, annealing at 50°C for 10 s, elongation at 72°C for 10 s, and a final extension at 72°C for 7 min. Three μL of PCR product was analysed by electrophoresis on a 1% (w/v) agarose gel. All samples were amplified in duplicate reactions, and duplicate PCR products were pooled and purified using CleanPCR (cleanNA, the Netherlands) according to the manufacturer's instructions. The DNA concentration of the purified amplicons was measured by Qubit (Thermo Fisher Scientific). The purified amplicons were pooled in equimolar amounts, including PCR products prepared from synthetic Mock communities of known composition as positive control and nuclease-free water as negative control, and sent for sequencing by Hiseq2000 (GATC-Biotech GmbH, now part of Eurofins Genomics Germany GmbH, Konstanz, Germany).

### Analysis of microbial community diversity

The raw sequence data was analysed by NG-Tax 2.0 (Poncheewin *et al*. 2019, Ramiro-Garcia *et al*. 2016), which consists of three core processes: barcode-primer filtering, amplicon sequencing variants (ASV) picking and taxonomic assignment. Only the reads completely pairing with primers and barcodes were retained. A threshold of 0.1% relative abundance was used on a per-sample basis to prevent the inclusion of spurious ASVs produced by sequencing and PCR errors. Taxonomic assignment was done based on Silva 132 SSU Ref (Edgar 2010, Yilmaz *et al*. 2014). The generated BIOM (Biological Observation Matrix) and tree files were further organised to form phyloseq objects (McMurdie and Holmes 2013). Downstream analyses, including alpha and beta diversity, and microbial composition were performed by Microbiome and Phyloseq R packages (Lahti and Shetty 2017, McMurdie and Holmes 2013).

### Statistical Analysis

Statistical analyses and graphics were performed in R (Team 2013) and the built-in libraries ggplot2, tidyverse, ggpubr and vegan. For microbial diversity analysis, alpha diversity was analysed using Phylogenetic diversity, Observed, Chao1, Shannon and Inverse Simpson indices. Student's t-test was used to assess significance of observed differences in alpha diversity indices. The function adonis as implemented in vegan (Oksanen *et al*. 2007) was used for permutational multivariate analysis of variance (PERMANOVA) to assess significance of observed differences in beta diversity based on unifrac distances (Lozupone and Knight 2005, Lozupone *et al*. 2011), in which the number of permutations was set to 999 by default. In addition, permutation tests for significance in CAPSCALE were also applied following the default settings.

## Results

### Reductive dechlorination of PCE in Aarhus Bay sediments

Samples from a sediment core taken in Aarhus Bay and depth-fractionated were incubated in mineral marine medium with a range of organohalides separately in the presence (S) and absence (NS) of sulfate (Table 1). Our results revealed the dehalogenating potential of Aarhus Bay sediments for various chlorinated, brominated and iodonated compounds. The microbial composition of PCE dechlorinating cultures exhibited evident differences to cultures to which other organohalides had been added (Fig. S1). Considering the discovery of *tceA*-like transcripts in sediments of Aarhus Bay (Zinke *et al*. 2017), PCE dechlorination was initially selected for subsequent experiments to demonstrate OHR in Aarhus Bay sediments, followed up by experiments with 2,6-DBP.

**Table 1.** List of halogenated compounds for testing reductive dehalogenation of Aarhus Bay marine sediments

Aarhus Bay Sediments^a^	Station	Halogenated Compounds	Sulfate Addition^b^	Products^c^
3–35 cm / 36–68 cm	M5	Tetrachloroethane (PCE)	+ / −	Trichloroethene (TCE)^e^; Cis-dichloroethene (cDCE)^f^;
3–35 cm / 36–68 cm	M5	2,6-Dibromophenol (2,6-DBP)	+ / −	Bromophenol (BP)^e^; Phenol^f^;
3–35 cm	M5	1,4-Dibromobenzene (1,4-DBB)	+ / −	Bromobenzene (BB)^e^; Benzene^f^;
3–35 cm	M5	3-Bromophenol (3-BP)	+ / −	Phenol^f^;
3–35 cm	M5	2,4,6-Trichlorophenol (2,4,6-TCP)	+ / −	ND^d^;
3–35 cm	M5	2,4,6-Triiodorophenol (2,4,6-TIP)	+ / −	4-Iodophenol (4-IP)^g^; Phenol^g^;

^a^ Different depths of Aarhus Bay marine sediments from one core.

^b^ Cultures incubated with (+) or without (−) sulfate.

^c^ Transformation products detected under both sulfate (+ / −) conditions.

^d^ ND means no dehalogenation.

^e^ Intermediate metabolites.

^f^ Final product after 100% disappearance of the original compound.

^g^ Indicates coexistance of metabolites 4-IP and phenol in a ratio around 1:2.

**Figure 2.**
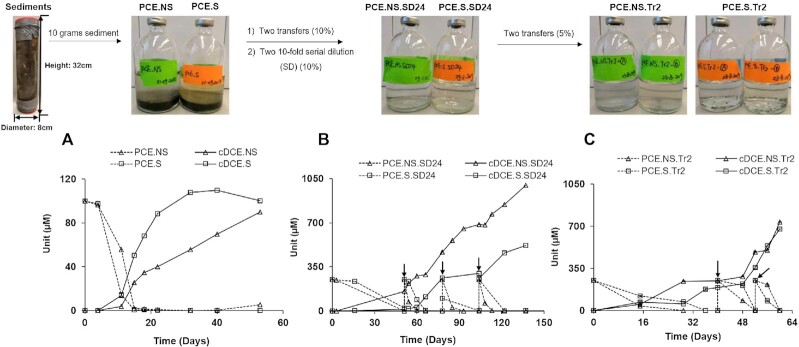
PCE dechlorination to cDCE in initial microcosms **(A)**, serial dilutions **(B)** and further transfers **(C)** in the presence (S) and absence (NS) of sulfate. SD.24:the fourth (i.e. 10^–4^ dilution) cultures in the second serial dilution in step 3 (Figure 1); Tr2: the second transfers in Step 4 (Figure 1). See also Figure 1 for a detailed scheme of the experimental set-up and history and relatedness of the different cultures. Vertical arrows indicate spikes of PCE. Data shown are average values, and error bars represent the standard deviation of the duplicates in Tr2. Error bars are not always visible due to small standard deviations.

Dechlorination of PCE to cDCE was observed with TCE as intermediate, within 15 days after inoculation with sediment obtained from Aarhus Bay (Fig. 2A). After two transfers and two consecutive serial dilution series, the obtained enrichments still actively dechlorinated PCE to cDCE (Fig. 1B). Thereinto, the most diluted dechlorinating cultures (the fourth cultures in the second serial dilution (NS/S.SD24)) were selected for two additional transfers aiming to enrich the responsible dechlorinating consortium. In the second transfer, cultures incubated in the presence (S) or absence (NS) of sulfate stably dechlorinated PCE to cDCE after three spikes of PCE (Fig. 2C). The overall experimental workflow is shown in Fig. 1, and results of transfers and serial dilutions are provided in Fig. S2 and Fig. S3, respectively.

### Detection of known and putative OHRB by quantitative PCR (qPCR) and 16S rRNA gene amplicon sequencing

**Figure 3.**
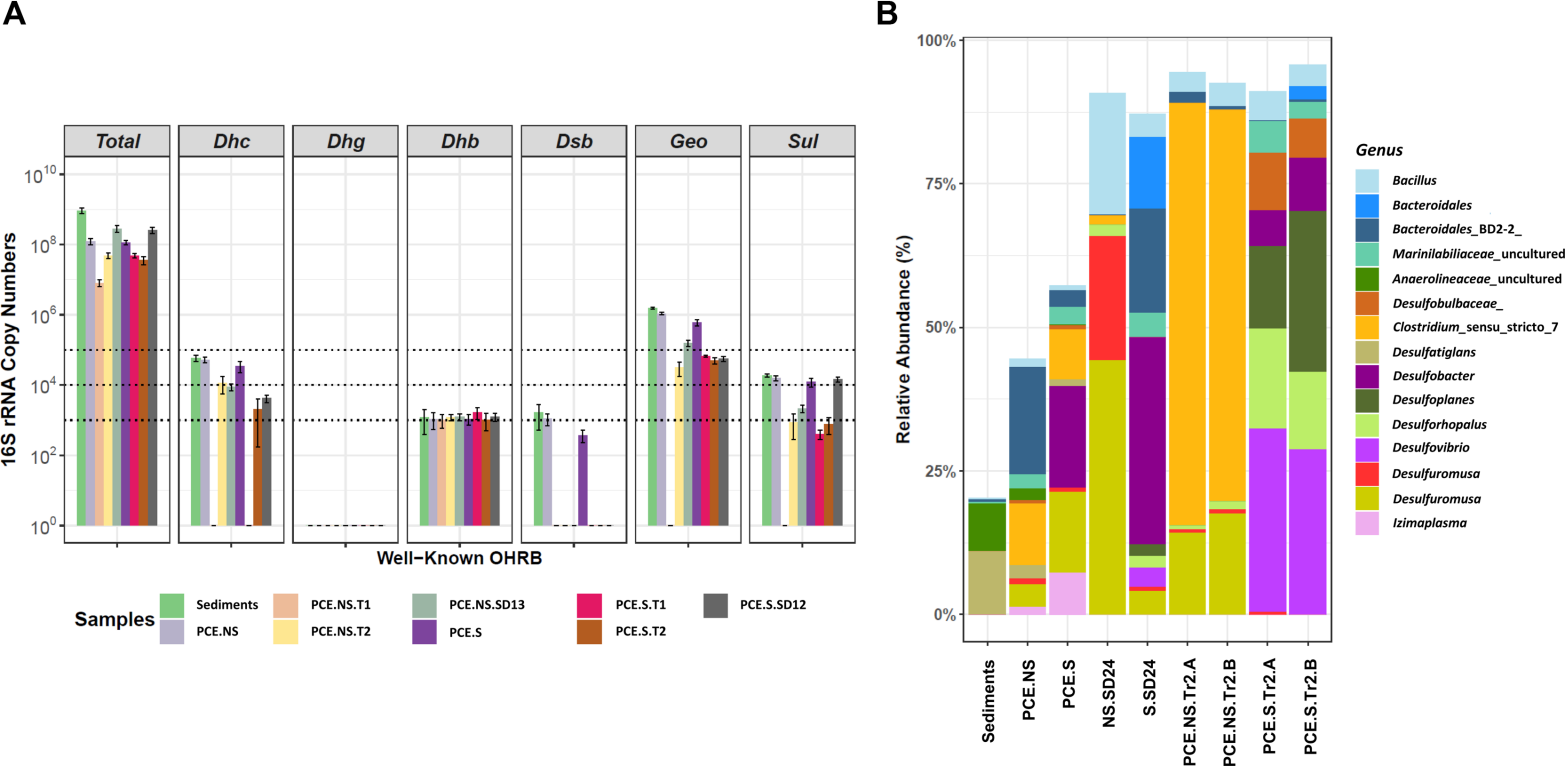
Microbial community analysis of PCE-dechlorinating cultures by qPCR**(A)** and 16S rRNA gene amplicon sequence data at genus level **(B)**. 16S rRNA gene copy numbers per millilitre of the cultures except for the marine sediments (Sediments) that are defined per gram of wet sediment. *Total*: total bacterial 16S rRNA gene copy numbers; *Dhc*: *Dehalococcoides*; *Dhg*: *Dehalogenimonas*; *Dhb*: *Dehalobacter*; *Dsb*: *Desulfitobacterium*; *Geo*: *Geobacter*; *Sul*: *Sulfurospirillum*. Values and error bars represent the averages and standard deviations of the triplicate qPCRs, respectively. Values below the detection limit are shown as one copy per ml. The added horizontal dotted lines represent 10^3^, 10^4^, and 10^5^ copy numbers, respectively. Only taxa that have a relative abundance > 5% in at least one of the samples are shown. Duplicate enrichments are indicated by suffixes ‘A’ and ‘B’. T1 and T2: the first and second transfers in step 2 (Figure 1); SD12 and SD13: the second (10^–2^) and third (10^–3^) dilution cultures in the presence and absence of sulfate, respectively, in the first serial dilution in step 3 (Figure 1). See also Figure S2.

qPCR analyses revealed the existence of well-known OHRB in the original sample, including *Dhc*, *Dhb*, *Dsb, Geo* and *Sul* ranging from 1.2 (0.8 SD) × 10^3^ of *Dhb* to 1.5 (0.1 SD) × 10^6^ of *Geo* per gram (Fig. 2A), which accounted for less than 1% of the total 16S rRNA gene copy numbers (9.2 (1.6 SD) × 10^8^). Based on 16S rRNA gene amplicon sequence data, relative abundances of these OHRB together were less than 2%, in line with the qPCR results. The 16S rRNA gene copy numbers and relative abundances of these well-characterized OHRB decreased during the enrichment procedure. In particular, *Dsb* was undetectable after the first serial dilution (Fig. 3A). For all samples, including original sediment fractions and derived cultures, the 16S rRNA gene of *Dehalogenimonas* (*Dhg*) and well-characterized RDase genes *vcrA*, *bvcA* and *tceA* were below the detection limit. Apparantly, the known OHRB were outcompeteted by other indigenous dehalogenators during PCE dechlorination. In contrast, the microbial community analysis indicated that certain bacteria, including *Halodesulfovibrio* in sulfate-free cultures, and *Desulfovibrio* in sulfate-amended cultures were enriched up to relative abundances of 16.0 (2.3 SD) % and 30.3 (1.7 SD) %, respectively, in Tr2.A/B cultures (Fig. 3B). Representative strains of both genera were recently shown to debrominate 2,6-DBP to phenol (Liu and Haggblom 2018). We therefore hypothesized that our enrichments might also possess the ability to debrominate 2,6-DBP to phenol, in line with results of the initial screening (Table 1).

### Switching the electron acceptor from PCE to 2,6-DBP

**Figure 4.**
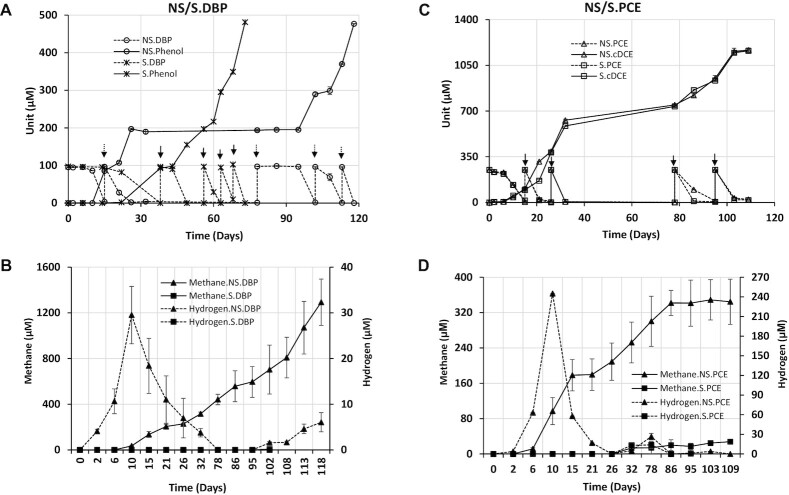
Reductive debromination of 2,6-DBP in addition to PCE dechlorination. In parallel cultures, 2,6-DBP and PCE are dehalogenated into phenol **(A)** and cDCE **(C)**, respectively. Methane and hydrogen **(B, D)** are measured throughout the experiment in the presence or absence of sulfate. Values and error bars shown in the figure are averages and standard deviation of duplicate cultures. Arrows indicate the spikes of PCE (black) and 2,6-DBP (grey-dotted arrows for sulfate-free cultures, black for sulfate-amended cultures).

As mentioned above, debromination of 2,6-DBP was observed in the initial screening of the dehalogenation potential of Aarhus Bay sediment. Moreover, members of enriched genera were previously reported to have debromination potential as outlined above. Thus, we tested whether the PCE dechlorinating enrichments retained the capacity of the original sediment for reductive debromination. Indeed, PCE-dechlorinating enrichments debrominated 2,6-DBP under both sulfate-free and sulfate-amended conditions. In contrast, the cultures failed to dechlorinate 2,6-DCP (Fig. 1), which was in agreement with the initial screening (Table 1). More detailed analyses of duplicate cultures revealed that 2,6-DBP was debrominated to phenol with bromophenol as intermediate (Fig. 4A). Five spikes of 100 µM 2,6-DBP each were introduced into the cultures. In parallel incubations, PCE dechlorination to cDCE was shown to be maintained as well (Fig. 4C).

Hydrogen was produced and accumulated up to 29.53 µM after 10 days in sulfate-free cultures after the first spike of 2,6-DBP (Fig. 4B). Then hydrogen was consumed alongside debromination, while methane accumulated after five spikes of 2,6-DBP up to 1.3 mM. In contrast, hydrogen and methane were not detected under sulfate-amended conditions (Fig. 4B). Lactate was utilized with the formation of propionate and acetate at an approximate ratio of 3:2.5 in sulfate-free cultures (Fig. S4A), whereas only acetate was produced and further utilized in sulfate-amended cultures (Fig. S4B). Similarly, hydrogen and methane were obviously produced in sulfate-free PCE-dechlorinating environments after the first 250 µM PCE spike, with particularly rapid hydrogen formation up to 245.13 µM (Fig. 4D). Notably, hydrogen and methane were produced in sulfate-amended cultures up to a detectable level after the third PCE spike, but only at concentrations below 30 µM. Lactate degradation followed a similar trend in PCE dechlorinating cultures as in the 2,6-DBP incubations (Fig. S4C&D). In all cultures where sulfate was added, this was reduced to sulfide.

### Microbial diversity and phylum-level composition of dehalogenating enrichments

**Figure 5.**
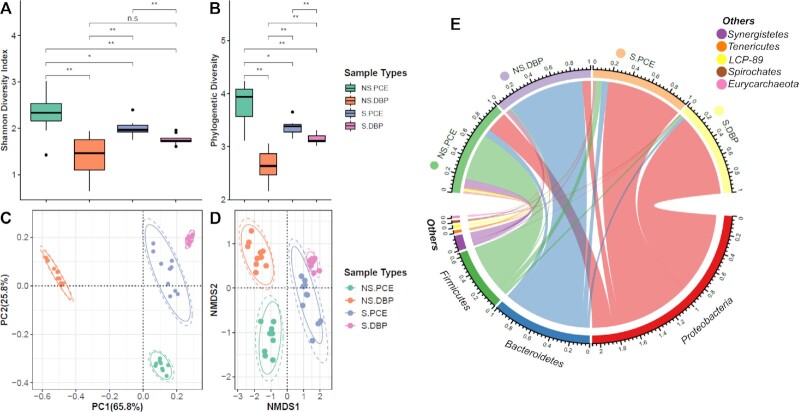
Analysis of microbial diversity. Alpha diversity analysis as based on Shannon index **(A)** and phylogenetic diversity **(B)**; beta diversity analysis including PCoA analysis of Weighted UniFrac distances **(C)**, and non-metric multidimensional scaling (NMDS) analysis of Bray–Curtis dissimilarity**(D)** with a stress value of 0.08. The average relative abundances at phylum level were calculated for the chord plot corresponding to the four sample types **(E)**. Duplicate cultures were included for each sample type, i.e. sulfate-free PCE dechlorination (NS.PCE, n = 9: 5 times sampling, duplicates, 1 failed (B1) as the purified amplicon in low quality), sulfate-free debromination (NS.DBP, n = 10: 5 times sampling, duplicates), sulfate-amended PCE dechlorination (S.PCE, n = 10: 5 times sampling, duplicates) and sulfate-amended debromination (S.DBP, n = 10: 5 times sampling, duplicates). Ellipses in C and D indicate the samples followed normal (dashed line) and t distribution (solid line) at a confidence level of 0.95. n.s: not significant, *P*-value > 0.05; “*”: 0.01 < *P*-value < 0.05; “**”: *P*-value < 0.01. PC1 and PC2 (C) are the first two principal components, with percentage of variation explained in parentheses.

To assess the microbial community structure in the different cultures, and particularly to compare PCE- and 2,6-DBP dehalogenating cultures, 16S rRNA gene amplicon sequencing was employed. ASV-based alpha diversity (Shannon index and phylogenetic distances) decreased significantly (*P* < 0.01) in 2,6-DBP debrominating cultures compared to the PCE dechlorinating cultures under the corresponding sulfate-free or sulfate-amended conditions (Fig. 5A & B). Beta diversity analysis using weighted Unifrac (W-Unifrac) distances revealed that the microbial community structure was clearly reshaped by changing the electron acceptor from PCE to 2,6-DBP, especially in the absence of sulfate (Fig. 5C). Further NMDS analysis was in agreement with W-Unifrac based ordination and displayed differences of microbial composition during debromination and dechlorination under sulfate-free or sulfate-amended conditions (Fig. 5D). Among all, sulfate-free debrominating cultures (NS.DBP) showed the biggest distance from the other three types, and the sulfate-amended cultures showed higher similarity regardless of the added organohalide (Fig. 5C and D). Further microbial composition analysis (Fig. 5E) showed that *Proteobacteria* was the predominant phylum accounting for 83 (6.6 SD) % and 93 (2.6 SD) % in sulfate-amended dechlorinating (S.PCE) and debrominating (S.DBP) cultures, respectively. *Firmicutes* and *Proteobacteria* were the main phyla in sulfate-free dechlorinating cultures (NS.PCE) accounting for 52 (16 SD) % and 23 (14 SD) %, respectively, whereas *Bacteroidetes* was predominant in sulfate-free debrominating cultures with 83 (5.1 SD) %.

### Microbial community dynamics at genus and ASVs levels

**Figure 6.**
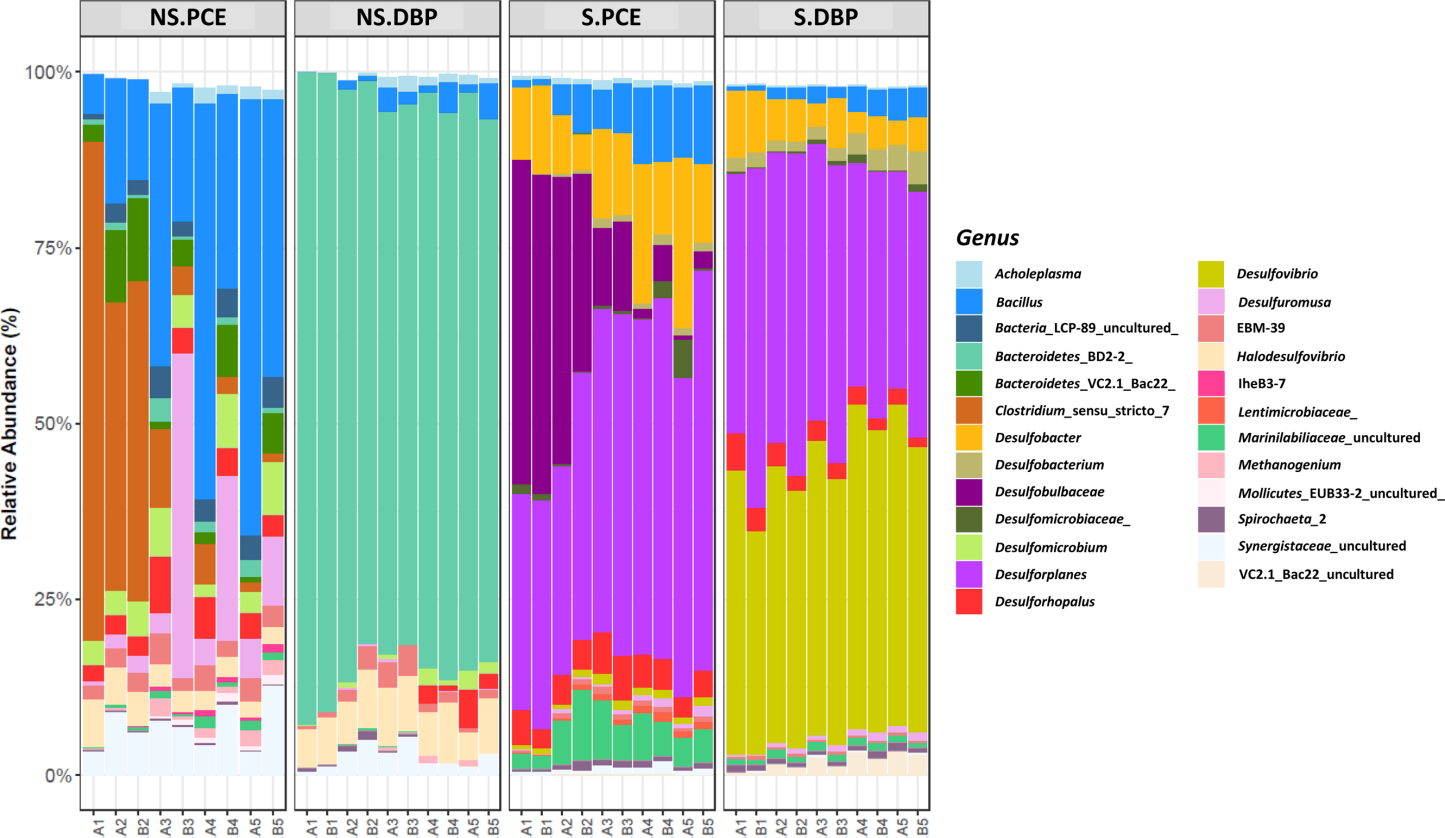
Dynamics of microbial community composition during reductive dehalogenation under sulfate-free (NS) and sulfate-amended (S) conditions. Relative abundance of microbial taxa is shown at genus level with a cut-off at 1% in at least one single sample. Samples are numbered from A1 to A5 and B1 to B5, indicating samples collected after each complete dehalogenation, prior to the spike of PCE or 2,6-DBP, respectively. B1 sampled from the sulfate-free PCE dechlorinating cultures (NS.PCE) was absent due to the low quality of the PCR product that did not meet the sequencing requirements.

**Figure 7.**
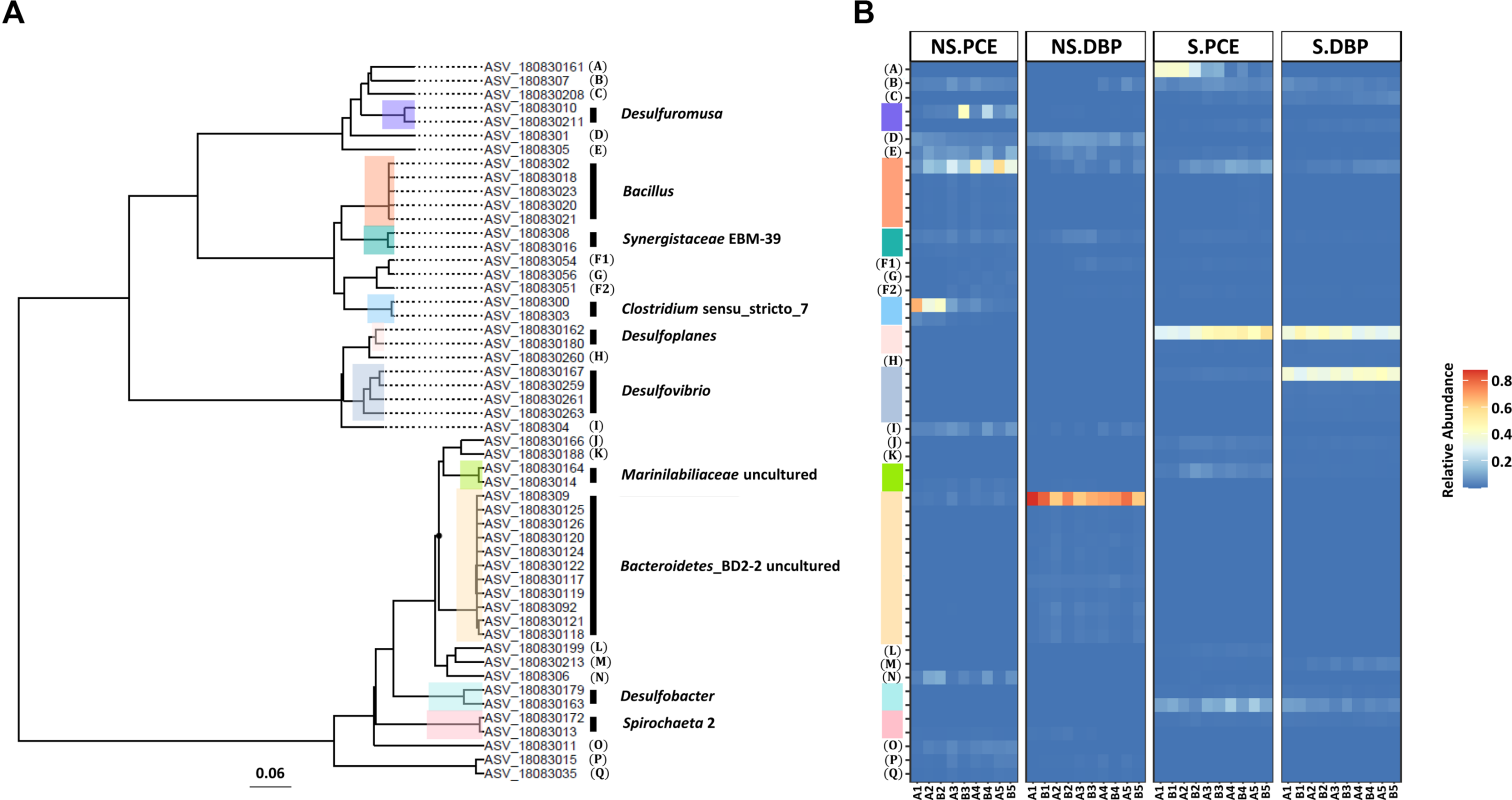
Phylogenetic analysis **(A)** and dynamics of microbial composition **(B)** at ASVs level. The cut-off relative abundance of ASVs is set at 0.1% in at least one single sample. ASVs belonging to the same genus are indicated by the same colour (top 10 genera) or letters. Alphabetic letters in Fig. 6A: A, *Desulfobulbaceae*_; B, *Desulforhopalus*; C, *Desulfobacterium*; D, *Halodesulfovibrio*; E, *Synergistaceae*_uncultured; F, *Acholeplasma*; G, *Mollicutes*_EUB33-2_uncultured_; H, *Desulfovibrionales*_; I, *Desulfomicrobium*; J, *Marinilabiliaceae*_; K, *Bacteroidetes*_vadinHA17_; L, *Lentimicrobiaceae*_; M, VC2.1_Bac22_uncultured_; N, VC2.1_Bac22_; O, Bacteria_LCP-89_; P, *Methanogenium*; Q, *Methanomicrobiaceae*_;

To elaborate in more detail on the microbial community changes associated with the enrichment of dechlorinating and debrominating cultures, comparisons were done at genus- and ASV level (Fig. 6 & 7). Most notably, we observed the predominance of an unknown genus from *Bacteroidetes*_BD2_2 in sulfate-free debrominating cultures (NS.DBP) accounting for 83 (5.2 SD) % in relative abundance (Fig. 6), which included 11 ASVs (Fig. 7A). Among these, ASV_1808309 accounted for > 50% of all reads in this genus (Fig. 7B). This was followed by *Halodesulfovibrio* enriched as the second-most predominant genus up to 6.9 (1.4 SD) %. In contrast, *Bacillus* was the predominant *genus* with a relative abundance of 31 (18 SD) % in sulfate-free dechlorinating cultures (NS.PCE), with ASV_18083010 and ASV_1808307 accounting for higher proportions than the other three ASVs (Fig. 7B), and *Halodesulfovibrio* accounted for a relative abundance of 3.7 (1.4 SD) % (Fig. 6). Notably, *Clostridium*_sensu_stricto_7 decreased in relative abundance from 70.9 to 1.3% after the 5^th^ addition of PCE in dechlorinating cultures in the absence of sulfate (Fig. 6), which was mainly caused by the decrease of ASV_1808300 (Fig. 7B). *Desulforhopalus* increased in relative abundance after the 4^th^ spike and up to 3.9% after the 5^th^ spike in debrominating cultures (NS.DBP), and was stably maintained in dechlorinating cultures (NS.PCE) at 4.1 (1.8 SD) % without sulfate addition. Similarly, *Desulfuromusa* showed an increasing trend in sulfate-free PCE dechlorinating cultures (NS.PCE) reaching up to 7.8 (2.1 SD) % after 5^th^ spike. Unlike the trend under sulfate-free conditions, *Desulfoplanes* became the major genus and was enriched up to 51 (5.8 SD) % after the 5^th^ PCE spike (S.PCE) in the presence of sulfate. Similarly, in debrominating cultures, *Desulfoplanes was observed at a stable and high* relative abundance of 38 (5.4 SD) % (S.DBP). In addition, *Desulfobacter* and *Bacillus* increased in sulfate-amended dechlorinating cultures (S.PCE) reaching up to 17.8% and 10.7% respectively. *Desulfovibrio* accounted for 40 (4.1 SD) % in sulfate-amended debrominating cultures (S.DBP), whereas it represented only a small proportion in sulfate-amended dechlorinating cultures (S.PCE) at 1.1 (0.28 SD) % (Fig. 6), which was the result of a lower relative abundance of ASV_180830167 (Fig. 7B). Overall, several *Desulfobacterota* taxa, including *Desulfobacter*, *Desulfobacterium*, *Desulfoplanes* and *Desulfovibrio* were only present in sulfate-amended cultures, whereas *Desulfomicrobium*, *Halodesulfovibrio* and *Methanogenium* were only presented under sulfate-free conditions.

## Discussion

Putative RDase genes are being increasingly reported from metagenomic analyses of marine sediments. Considering the global distribution of organohalides in marine environments and their excellent potential as electron acceptors for anaerobic respiration, it is tempting to assume that OHR is occurring in Aarhus Bay sediments contributing to recycling halides, carbon and other nutrients. However, experimental verification of OHR potential in these sediments usually has not been achieved. Recent metagenomic and single-cell genomic analysis revealed presence of putative RDase genes in Aarhus Bay sediments, indicating the possibility for alternative energy conservation through reductive dehalogenation in addition to sulfate reduction at the near-surface sulfate-rich zone (Jochum *et al*. 2017, Jochum *et al*. 2018). On this basis, we were inspired to investigate OHR potential and the eco-physiology of associated microbial communities.

### Reductive dehalogenation of various organohalides by Aarhus Bay marine sediments

Similar to former studies of reductive dehalogenation in marine environments (Ahn *et al*. 2003, Futagami *et al*. 2009, Futagami *et al*. 2013, Kaster *et al*. 2014, Matturro *et al*. 2016), we could show that pristine marine sediments from Aarhus Bay could reductively dehalogenate a range of organohalides, including PCE, 2,6-DBP, 1,4-DBB, 3-BP and 2,4,6-TIP (Table 1), suggesting the presence of multiple reductive dehalogenating microorganisms and corresponding RDase genes. *Firmicutes* were enriched in all dehalogenating cultures (Fig. S1), with members of the *Clostridiales* being most predominant. These populations may serve as syntrophic partner to dehalogenating populations, for example as hydrogen producer as has been shown previously (Lin *et al*. 2020, Merlino *et al*. 2015, Yang *et al*. 2019). Intriguingly, *Lokiarchaeia* belonging to Asgard archaea (Spang *et al*. 2017, Zaremba-Niedzwiedzka *et al*. 2017), a recently described group of archaea linked to the origin of eukaryotes (Eme *et al*. 2017), were observed in the original sediment and enriched only in 2,4,6-TIP deiodinating cultures regardless of sulfate addition. To this end, it seems promising to employ reductive deiodination to enrich or even isolate strains from *Lokiarchaeota*, previously shown to bear putative RDase genes (Spang *et al*. 2019). In addition, *Anaerolineae*, belonging to *Chloroflexi*, were sharply decreased in PCE dehalogenating cultures compared to incubations with other organohalides. Recently, metagenome assembled genomes (MAGs) revealed that *Anaerolineae* have the potential of reductive dehalogenation (Fincker *et al*. 2020), however, this has not been experimentally confirmed to date. Finally, members of the *Bacteroidetes* were enriched only in PCE dechlorinating cultures. Considering that to date there are no representative OHRB identified from this phylum, results presented here provide new leads for the isolation and characterization of yet unknown organohalide respiring prokaryotes.

### Microbial composition of PCE dechlorination enrichments

Populations of well-characterized OHRB including *Dhc*, *Dhb*, *Dsb*, *Geo*, and *Sul* (Adrian and Loeffler 2016, Atashgahi *et al*. 2016, Atashgahi *et al*. 2013, Azizian *et al*. 2010a, Fincker and Spormann 2017) were found in the marine sediment studied here, but only at a low abundance below 10^6^ 16S rRNA gene copies/gram sediment. These microbes further decreased in abundance during transfers and serial dilutions indicating they are unlikely responsible for the PCE dechlorination under the conditions used in this study. We also did not detect known genes encoding the enzymes responsible for reductive dehalogenation of PCE and its metabolites, i.e. *vcrA-*, *bvcA-*, and *tceA* genes (Chen *et al*. 2014, Smits *et al*. 2004), suggesting the presence of other, variant sequences of RDase genes as previously indicated in subseafloor sediments (Futagami *et al*. 2013). The lack of dechlorination beyond cDCE suggests that enzymes required for cDCE and VC dehalogenation were not enriched under the conditions used here as previously observed for other environments (Krajmalnik-Brown *et al*. 2004, Scheutz *et al*. 2008). This is a common finding indicating likely evolution of VC-respiring *Dehalococcoidia* members due to anthropogenic contaminations in terrestrial environments (McMurdie *et al*. 2009). Microbial community analysis revealed that the original Aarhus Bay sediment sample contained two predominant genera with relative abundances over 5% (Fig. 3B), including an uncultured genus in the *Anaerolineaceae* belonging to the *Chloroflexi*, and the *Desulfobacterota* genus *Desulfatiglans*. Recent metagenomic data analysis revealed that members of the class *Anaerolineae* encoded putative RDase genes in their genomes, suggesting they might have the potential of reductive dehalogenation (Fincker *et al*. 2020). Furthermore, single-cell genomics has shown that *Desulfatiglans*-associated microorganisms contain putative RDase genes in their genomes (Jochum *et al*. 2018). However, in contrast to canonical RDases, the encoded putative RDase proteins contain transmembrane domains at the N-terminus but lacked a TAT signal peptide. Also, no accompanying RDase *B* gene was observed that normally encodes the membrane anchor for the catalytic subunit of RDases (Jochum *et al*. 2018). These putative RDases termed as hybrid RDases have not been shown to be functional (Atashgahi 2019). Intriguingly, the relative abundance of *Anaerolineaceae* and *Desulfatiglans* decreased to less than 1% in our highly enriched cultures regardless of the presence of sulfate suggesting they might not be responsible for the observed dehalogenation. In contrast, *Clostridium*_sensu_stricto_7 belonging to the *Firmicutes*, and the *Desulfobacterota* genus *Halodesulfovibrio* were enriched in sulfate-free cultures, whereas other members of the *Desulfobacterota* including *Desulfovibrio*, *Desulforhopalus*, *Desulfoplanes* and an unknown genus in the *Desulfobulbaceae* became the predominant genera in sulfate-amended cultures (Fig. 3B & Fig. S3B). To our knowledge, *Clostridium* members have not been shown to mediate OHR and have been proposed to function as the hydrogen producers facilitating reductive dehalogenation (Lin *et al*. 2020, Lo *et al*. 2020, Yang *et al*. 2019). In contrast, *Desulfobacterota* representatives, including *Halodesulfovibrio marinisediminis* and *Desulfovibrio bizertensis*, have been shown to debrominate 2,4,6-tribromophenol and 2,6-DBP into 4-bromophenol (4-BP) and phenol, respectively (Liu and Haggblom 2018). Furthermore, the genome of *Desulforhopalus singaporensis* was annotated to contain putative RDase genes (GenBank Accession: GCA_900104445.1), but OHR potential in this bacterium has not been experimentally verified (Lie *et al*. 1999). Henceforth, we speculated that the well-identified OHRB might be outweighed by other potential dehalogenating microbes in our laboratory microcosms.

### Switching organohalides from PCE to 2,6-DBP

The sediments of Aarhus Bay were previously reported to have a high Br/Cl ion ratio, and a variety of brominated organic compounds have been identified, implying the potential for debromination may exist in the seafloor sediments (Christensen and Platz 2001, Jorgensen *et al*. 2020, Zinke *et al*. 2017). In addition, our initial assessment of dehalogenation capacity corroborated debrominating potential of Aarhus Bay sediments. Further, the genera enriched in PCE-dechlorinating cultures (Fig. 2), like *Desulfovibrio*, have previously been reported to include strains that were characterized to debrominate 2,4,6-TBP and 2,6-DBP (Liu and Haggblom 2018). Interestingly, a recent study revealed the possibility of dechlorinating cultures to catalyze debromination (Xu *et al*. 2022). Indeed, our results are in agreement, as PCE-dechlorinating cultures showed the potential for 2,6-DBP debromination to phenol, whereas 2,6-DCP was not dechlorinated (Fig. 1). Similarly, Peng *et al* found marine *Desulfoluna* strains were capable of reductive debromination but not reductive dechlorination (Peng *et al*. 2020a). This may indicate niche specialization of marine OHRB for reductive debromination that could gain more energy to support bacterial growth than that of reductive dechlorination (Xu *et al*. 2022). The formation and consumption of hydrogen was observed in sulfate-free cultures (Fig. 4), indicating that hydrogen likely served as the intermediate electron donor for OHR and methanogenesis (Azizian *et al*. 2010a, Dolfing and Tiedje 1987) that occurred simultaneously as formerly reported (Aulenta *et al*. 2002). On the contrary, methane was not generated in sulfate-amended cultures at first that might be due to the fact that sulfate-reducing bacteria outcompeted methanogens due to higher substrate affinity of sulfate-reducing bacteria to hydrogen than methanogens (Kristjansson and Schönheit 1983, Piché-Choquette and Constant 2019). With the sulfate reduced, methane was detected but at a low concentration below 30 µM (Fig. 4B&D). Interestingly, reductive dechlorination and debromination were not influenced by the presence of sulfate, which was in line with recent reports of marine OHRB belonging to sulfate-reducing bacteria (SRB) (Liu *et al*. 2020, Liu and Haggblom 2018, Peng *et al*. 2020a). It is likely that the marine OHRB have developed strategies for concurrent sulfate and organohalide respiration.

### Potential OHRB inferred from microbial community analysis

Our results revealed that microorganisms that were previously discovered by metagenome and single-genome assembly as candidate OHRB, such as *Desulfatiglans* and its relatives (Fincker *et al*. 2020, Jochum *et al*. 2018), were not enriched in PCE dechlorination cultures, which might due to their inability to dechlorinate PCE under the conditions used for the experiments here (Fig. 3 and Fig. S3). Furthermore, some of the functionally characterized OHRB, for instance *Dehalococcoides*, that have been observed in Aarhus Bay sediments based on metagenomic analysis (Fincker *et al*. 2020), and which strictly depend on hydrogen as electron donor and halogenated compounds as electron acceptor for energy conservation (Maymo-Gatell *et al*. 1997), were not enriched in our cultures. Their fastidious and restricted metabolism might have rendered them less competitive, being outcompeted by other, more versatile, OHRB, like RDase-containing sulfate reducers (Peng *et al*. 2020a), in the defined mineral marine medium used in this study (Monserrate and Häggblom 1997). Changing the organohalide electron acceptors significantly reshaped the microbial community structure (Figs 5 and 6), suggesting that different microorganisms might be involved in the dehalogenation of the different chlorinated and brominated compounds tested in our study. Furthermore, the observed decrease in alpha diversity in debrominating cultures suggests that 2,6-DBP or the debrominated phenol might inhibit growth of certain bacteria via the leakage of cellular components, such as K^+^ and ATP, or even cell membrane destruction (Cooper *et al*. 2015, Escher *et al*. 1996, Heipieper *et al*. 1991, Stasiuk and Kozubek 2008). Intriguingly, transfers of cultures from the initial screening able to dehalogenate 2,6-DBP were no longer able to debrominate 2,6-DBP. As the microbial community analysis revealed the apparent loss of *Desulfovibrio* in comparison to the transfers from sulfate-amended dechlorinating cultures (Fig. S1 and S5), a likely role of *Desulfovibrio* for reductive debromination can be hypothesized. Interestingly, members of the genus *Bacillus* were strongly enriched in sulfate-free dechlorinating cultures. This genus has to date not been characterized to perform reductive dehalogenation. Interestingly, Lim *et al*. reported that members of the phylum *Bacteroidetes* were enriched in the presence of natural organohalides (Lim *et al*. 2018). Similarly, we observed that members of this phylum were also enriched in sulfate-free debrominating cultures, but OHRB belonging to *Bacteroidetes* remain uncharacterized, providing leads for future attempts to isolate these organisms.

Furthermore, members of several genera recently identified as OHRB were observed, including *Desulfuromusa*, *Halodesulfovibrio* and *Desulfovibrio* (Liu and Haggblom 2018). For each of these genera, several species-level amplicon sequencing variants (ASVs) were observed suggesting that the enriched populations were composed of more than one strain type, such as *Desulfuromusa* with two ASVs (Fig. 7). To this end, future studies should aim to provide strain-resolved information by meta-omics, such as metagenomics and meta-transcriptomics. Genetic information of new OHRB can be disclosed from genome-resolved binning of metagenome data, providing leads regarding metabolic differences that can guide efforts towards isolation and further characterization of yet unknown dehalogenators.

## Conclusions

In conclusion, this work verified the potential for OHR in Aarhus Bay sediments that have previously been shown to be a source of organohalides and putative RDase genes. This is of importance considering the increasing number of studies reporting occurrence of organohalides and putative RDase genes in marine sediments (Fincker *et al*. 2020, Jorgensen *et al*. 2020, Peng *et al*. 2020a, Peng *et al*. 2020b). Considering the diversity of organohalides naturally produced in marine environments, OHR should play a key role in recycling halides and organic carbon back to the seawater.

## Data availability

The nucleotide sequence data has been deposited in the European Bioinformatics Institute under accession number PRJEB50583.

## Supplementary Material

fiac073_Supplemental_File
