# Supplementary material for: Organohalide respiration potential in marine sediments from Aarhus Bay
Source: FEMS Microbiol Ecol. 2022 Jun 11;98(8):fiac073. doi: 10.1093/femsec/fiac073 (PMC9303371; doi:10.1093/femsec/fiac073)
Supplement: fiac073_Supplemental_File [file fiac073_supplemental_file.docx]

**Supplementary Data**

**Organohalide Respiration Potential in Marine sediments from Aarhus Bay**

Chen Zhang^1,2*^, Siavash Atashgahi^1^, Tom N.P Bosma^1^, Peng Peng^1,3^, Hauke Smidt^1*^

^1^Laboratory of Microbiology, Wageningen University & Research, Wageningen 6708 WE, The Netherlands

^2^[Biotechnology Research Institute, Chinese Academy of Agricultural Sciences, Beijing 100081, PR China](https://www.microbiologyresearch.org/search?option1=pub_affiliation&value1=%22Biotechnology+Research+Institute%2C+Chinese+Academy+of+Agricultural+Sciences%2C+Beijing+100081%2C+PR+China%22&option912=resultCategory&value912=ResearchPublicationContent)

^3^Current address: Department of Civil and Environmental Engineering, University of Michigan, Ann Arbor, Michigan 48109-2125, USA

∗Corresponding authors: Laboratory of Microbiology, Wageningen University & Research, Stippeneng 4, 6708 WE Wageningen, The Netherlands. Tel: +31657492180; E-mail: [chen3.zhang@wur.nl](mailto:chen3.zhang@wur.nl)

Laboratory of Microbiology, Wageningen University & Research, Stippeneng 4, 6708 WE Wageningen, The Netherlands. Tel: +31317483102; E-mail: [hauke.smidt@wur.nl](mailto:hauke.smidt@wur.nl)


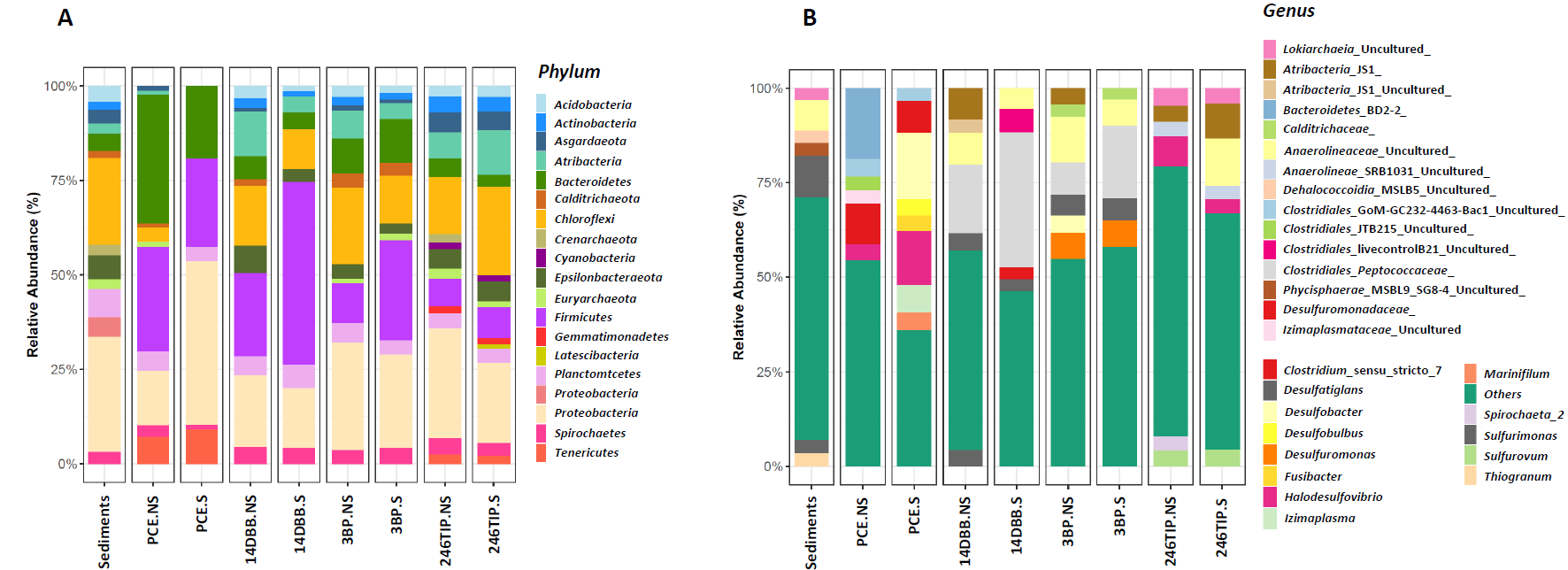


Figure S1. Microbial composition of various organohalide-respiring cultures under sulfate-free (NS) and sulfate-amended (S) conditions. Microbial community is analyzed at Phylum (A) and Genus (B) level. The cut-off relative abundance is set at 1 % for phylum and 3 % for genus. “*Others*” at genus level summarizes all taxa that are below the cut-off threshold (3 %). NS: non-sulfate; S: sulfate-amended; 14DBB: 1,4-dibromobenzene; 3BP: 3-bromophenol; 246TIP: 2,4,6-Triiodophenol.


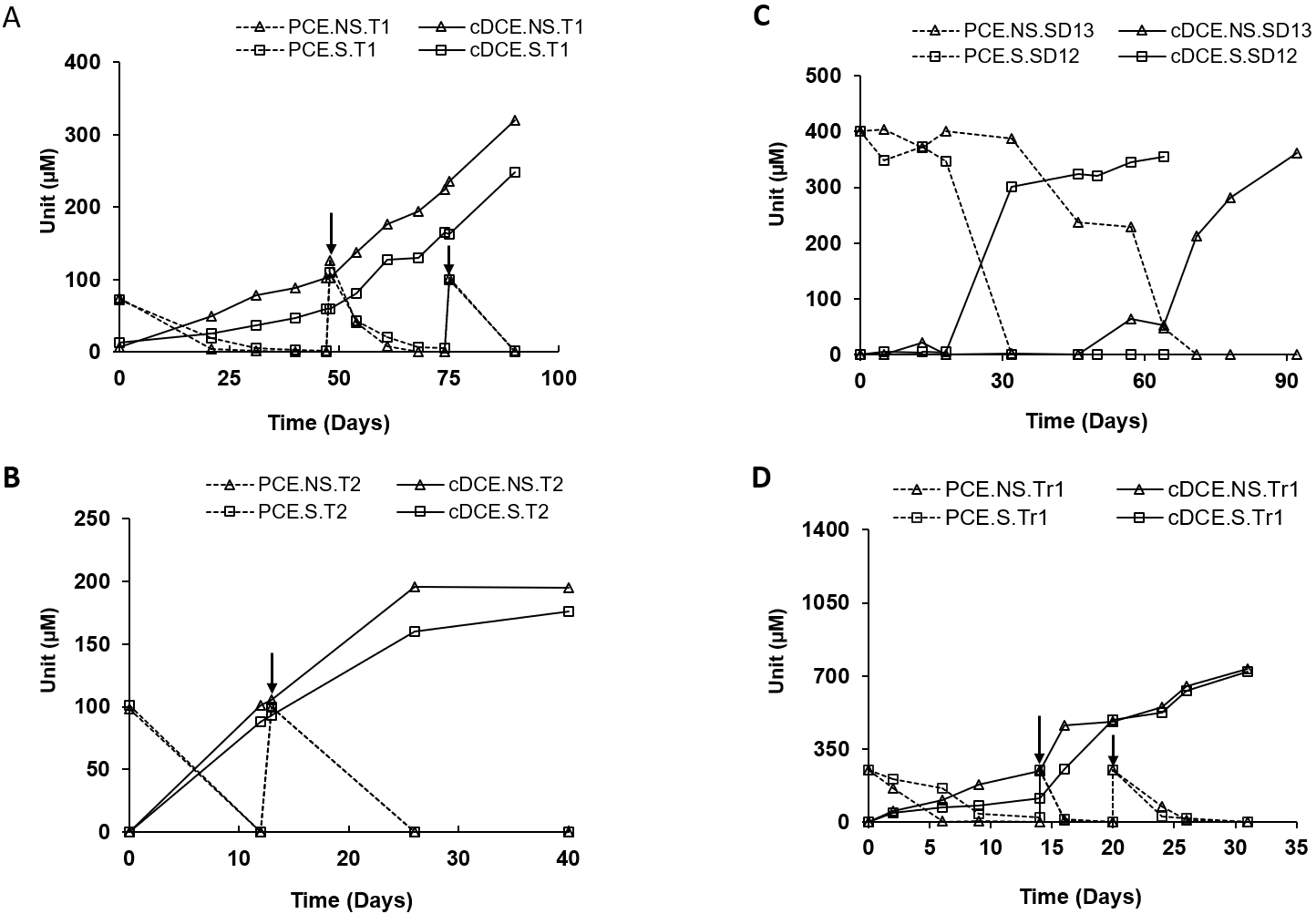


Figure S2. PCE dechlorinating cultures of 1^st^ two-time transfers (A: T1 and B: T2), 1^st^ serial dilution (C: SD13 and SD12) and 2^nd^ two-time transfers (D: Tr1). PCE.NS.SD13: the third bottle (10^-3^) as the highest dilution to maintain PCE dechlorination in the absence of sulfate in the first serial dilution. PCE.S.SD12: second bottle (10^-2^) as the highest dilution showing PCE dechlorination under sulfate-amended conditions. Tr1: First of the second two-time transfers. The arrows indicate spikes of PCE as well as lactate (NS) or lactate and sulfate (S).


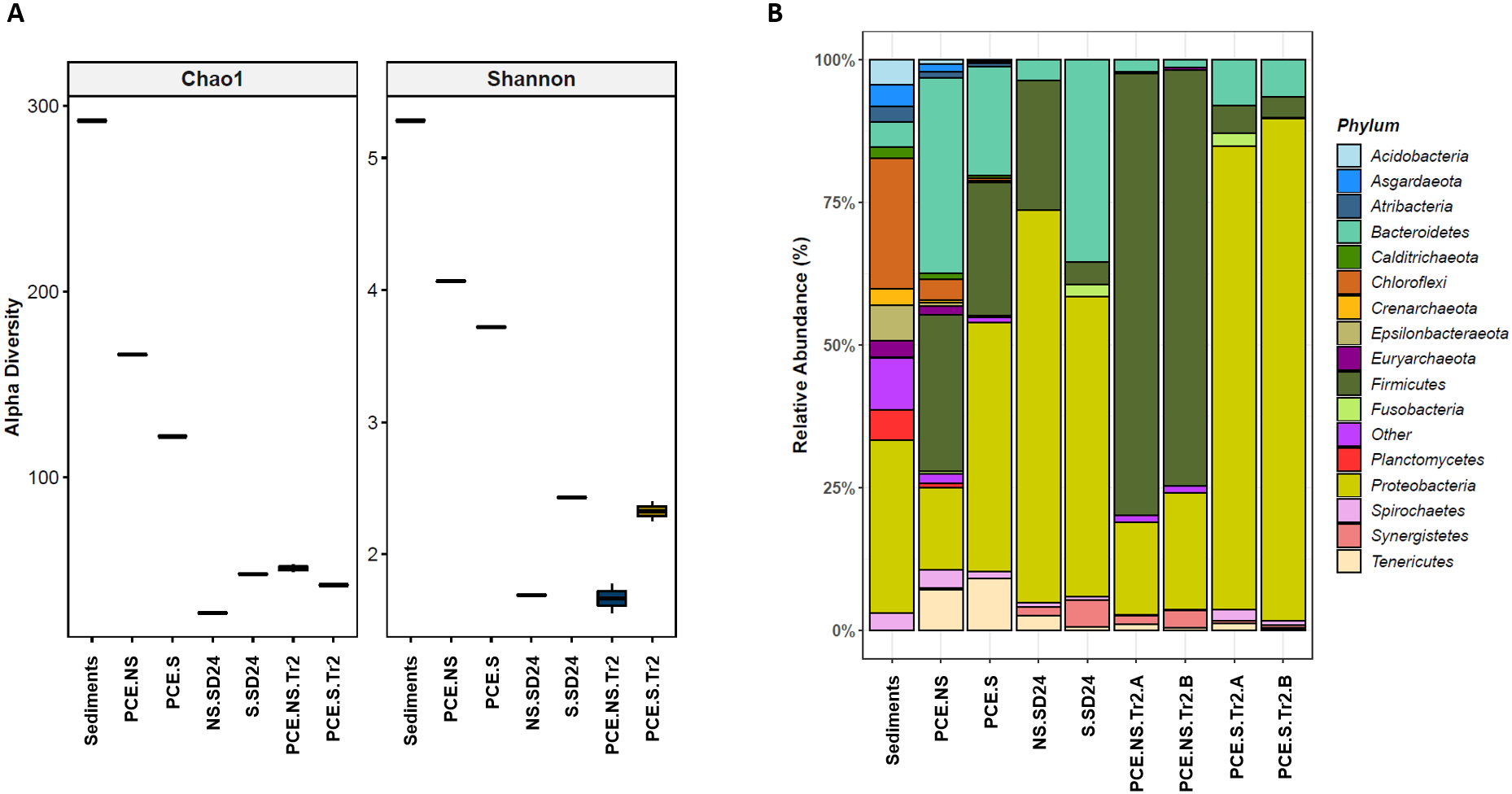


Figure S3. Alpha diversity analysis based on Chao1 and Shannon index (A) and microbial composition of PCE dechlorinating cultures at phylum level (B). Top 16 phyla are shown. Remaining phyla are shown as “other”.


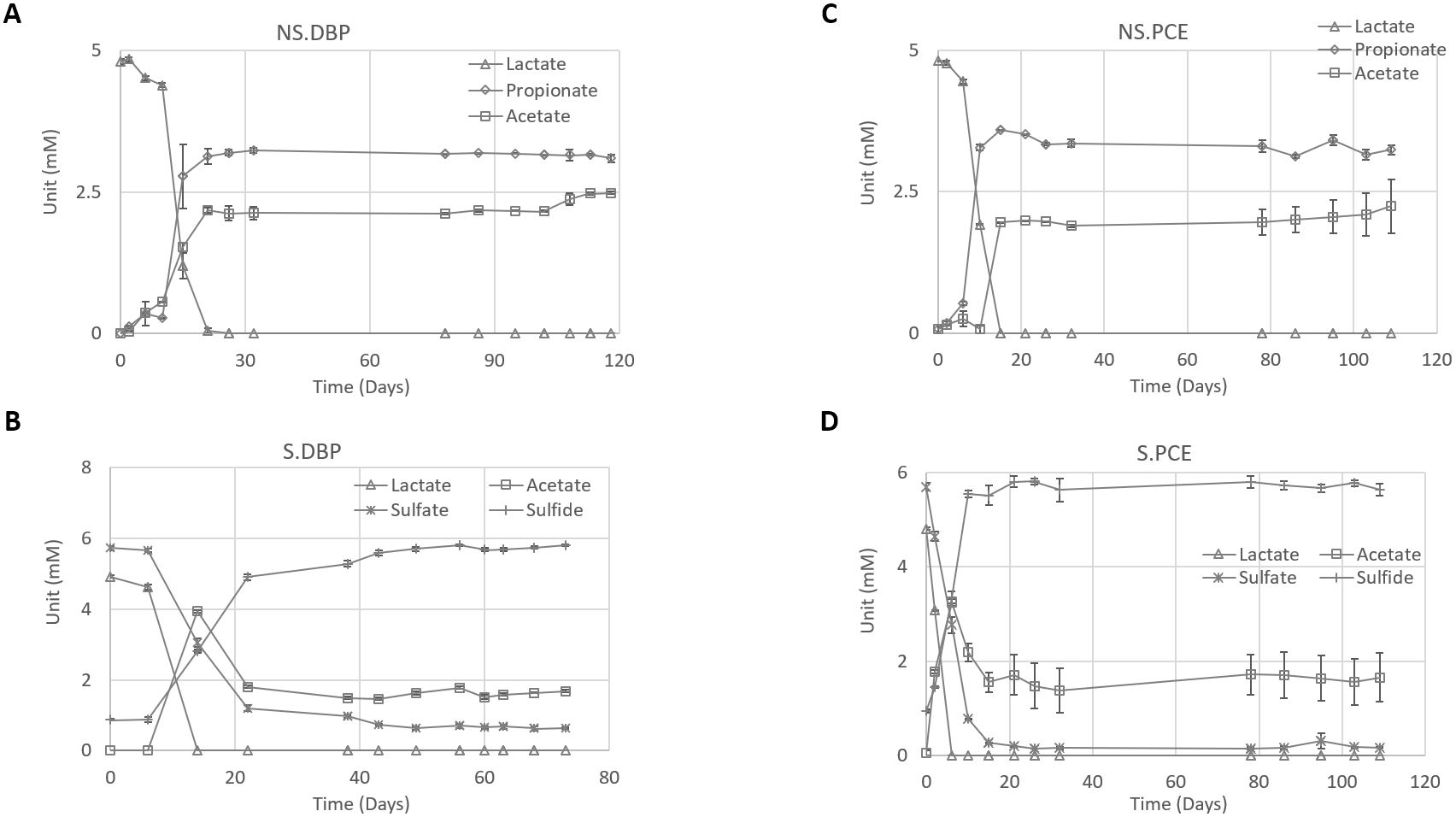


Figure S4. Metabolite concentrations in bottles amended with PCE (A,B) and 2,6-DBP (C,D) under sulfate-free (NS) and sulfate-amended (S) conditions. “A” and “B” represent duplicate bottles. Data is shown as average values with standard deviation from duplicate cultures.


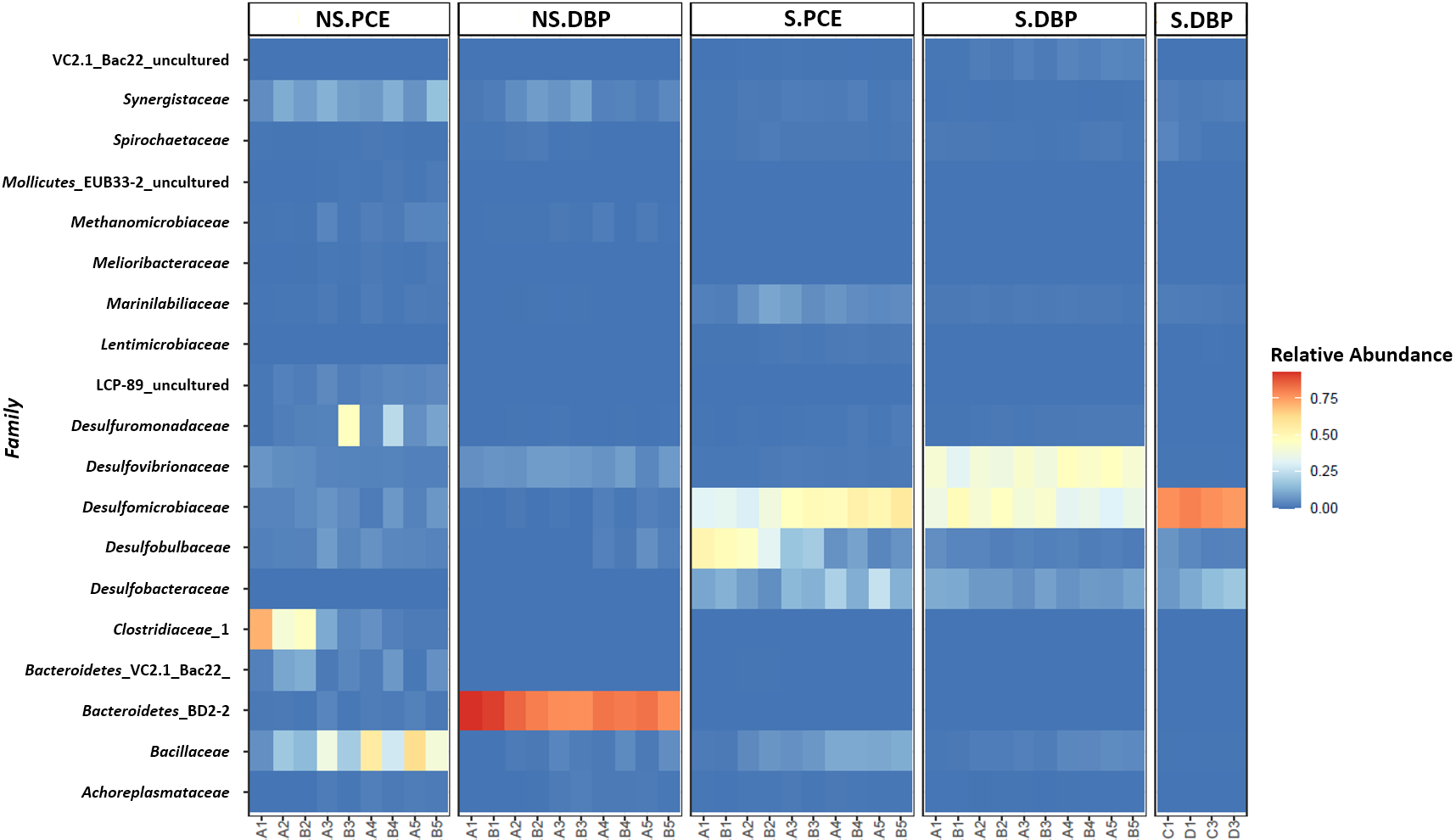


Figure S5. Dynamics of microbial composition at family level of four different cultures (NS_PCE, sulfate-free PCE dechlorination; NS_DBP, sulfate-free debromination; S_PCR sulfate-amended PCE dechlorination; S_DBP, sulfate-amended debromination). The cut-off of relative abundance was set at 1%. The duplicate cultures, S.DBP.CD, unable to debrominate 2,6-DBP, were included.

Table S1. Primers used in this study. All primers target the 16S rRNA gene, except for *vcrA* and *bvcA*

that target reductive dehalogenase genes.

| Target | Name | Oligonucleotide sequence (5´–3´) | Reference for primer | Reference for qPCR program |
| --- | --- | --- | --- | --- |
| Bacteria | Eub341F | CCTACGGGAGGCAGCAG | (Muyzer*, et al.* 1993) | (Atashgahi*, et al.* 2013) |
|  | Eub534R | ATTACCGCGGCTGCTGGC |  |  |
|  |  |  |  |  |
| *Desulfitobacterium* | Dsb406F | GTACGACGAAGGCCTTCGGGT | (Smits*, et al.* 2004) | (Smits*, et al.* 2004) |
|  | Dsb619R | CCCAGGGTTGAGCCCTAGGT |  |  |
|  |  |  |  |  |
| *Dehalococcoides* | Dco728F | AAGGCGGTTTTCTAGGTTGTCAC | (Smits*, et al.* 2004) | (Atashgahi*, et al.* 2013) |
|  | Dco944R | CTTCATGCATGTCAAAT |  |  |
|  |  |  |  |  |
| *Dehalobacter* | Dre441F | GTTAGGGAAGAACGGCATCTGT | (Smits*, et al.* 2004) | (Atashgahi*, et al.* 2013) |
|  | Dre645R | CCTCTCCTGTCCTCAAGCCATA |  |  |
|  |  |  |  |  |
| *Dehalogenimonas* | BL-DC-1243F  BL-DC-1351R | GGYACAATGGGTTGCCACCGG  AACGCGCTATGCTGACACGCGT | (Chen*, et al.* 2014) | (Chen*, et al.* 2014)^a^ |
|  |  |  |  |  |
| *Geobacter* | Geo196F | GAATATGCTCCTGATTC | (Amos*, et al.* 2007) | (Azizian*, et al.* 2010) |
|  | Geo535R | TAAATCCGAACAACGCTT |  |  |
|  |  |  |  |  |
| *Sulfurospirillum* | Sulfuro114F | GCTAACCTGCCCTTTAGTGG | (Sutton*, et al.* 2015) | (Sutton*, et al.* 2015) |
|  | Sulfuro421R | GTTTACACACCGAAATGCGT |  |  |
| *tceA* | TceA1270F  TceA 1336R | ATCCAGATTATGACCCTGGTGAA  GCGGCATATATTAGGGCATCTT | (Ritalahti*, et al.* 2006) | (Ritalahti*, et al.* 2006) |
| *vcrA* | Vcr1022F  Vcr1093R | CGGGCGGATGCACTATTTT  GAATAGTCCGTGCCCTTCCTC | (Ritalahti*, et al.* 2006) | (Ritalahti*, et al.* 2006) |
|  |  |  |  |  |
| *bvcA* | Bvc925F  Bvc1017R | AAAAGCACTTGGCTATCAAGGAC  CCAAAAGCACCACCAGGTC | (Ritalahti*, et al.* 2006) | (Ritalahti*, et al.* 2006) |

^a^ The qPCR program was modified as 98°C for 5 min, followed by 40 cycles of 98°C for 15 s, 68.2°C for 45 s. Melting curves were included from 55°C to 95°C with increments of 0.5°C and 10 s at each step.

**References**

Amos BK, Sung Y, Fletcher KE *et al.* Detection and quantification of *Geobacter lovleyi* strain SZ: implications for bioremediation at tetrachloroethene-and uranium-impacted sites. *Appl Environ Microbiol* 2007;73: 6898-904.

Atashgahi S, Maphosa F, Doğan E *et al.* Small-scale oxygen distribution determines the vinyl chloride biodegradation pathway in surficial sediments of riverbed hyporheic zones. *FEMS Microbiol Ecol* 2013;84: 133-42.

Azizian MF, Marshall IP, Behrens S *et al.* Comparison of lactate, formate, and propionate as hydrogen donors for the reductive dehalogenation of trichloroethene in a continuous-flow column. *J Contam Hydrol* 2010;113: 77-92.

Chen J, Bowman KS, Rainey FA *et al.* Reassessment of PCR primers targeting 16S rRNA genes of the organohalide-respiring genus *Dehalogenimonas*. *Biodegradation* 2014;25: 747-56.

Muyzer G, De Waal EC, Uitterlinden AG. Profiling of complex microbial populations by denaturing gradient gel electrophoresis analysis of polymerase chain reaction-amplified genes coding for 16S rRNA. *Appl Environ Microbiol* 1993;59: 695-700.

Ritalahti KM, Amos BK, Sung Y *et al.* Quantitative PCR targeting 16S rRNA and reductive dehalogenase genes simultaneously monitors multiple *Dehalococcoides* strains. *Appl Environ Microbiol* 2006;72: 2765-74.

Smits TH, Devenoges C, Szynalski K *et al.* Development of a real-time PCR method for quantification of the three genera *Dehalobacter,* *Dehalococcoides*, and *Desulfitobacterium* in microbial communities. *J Microbiol Methods* 2004;57: 369-78.

Sutton NB, Atashgahi S, Saccenti E *et al.* Microbial community response of an organohalide respiring enrichment culture to permanganate oxidation. *PLoS One* 2015;10: e0134615.
